# Spontaneous Rectus Sheath Hematoma

**DOI:** 10.7759/cureus.44138

**Published:** 2023-08-25

**Authors:** Snehasis Das, Sagar Prakash, Shweta Singh, Oseen Shaikh, Gopal Balasubramanian

**Affiliations:** 1 Surgery, Jawaharlal Institute of Postgraduate Medical Education and Research, Puducherry, IND; 2 Radiodiagnosis, Jawaharlal Institute of Postgraduate Medical Education and Research, Puducherry, IND

**Keywords:** angioembolization, profundal femoris artery, inferior epigastric artery, digital subtraction angiography, rectus sheath hematoma

## Abstract

Rectus sheath hematoma (RSH) is one of the surgical emergencies that mimics peritonitis or other causes of acute abdominal pain. It is usually seen in old age, post-trauma, anticoagulation therapy pregnancy, chronic cough, and liver disease. Nevertheless, RSHs can be spontaneous without any underlying predisposing factors. Here, we present a 51-year-old female with sudden onset abdominal pain, abdominal distention, hypotension, and severe pallor. After initial resuscitation, the patient underwent radiological imaging. This suggested an RSH with active bleeding from the inferior epigastric artery or profunda femoris artery. The patient underwent digital subtraction angiography and angioembolization of the profunda femoris branch. After a few days, the patient continued deteriorating and succumbed to acute respiratory distress syndrome (ARDS).

## Introduction

Rectus sheath hematoma (RSH) is rare and usually occurs due to anticoagulation therapy, trauma, or inherent bleeding diathesis [1]. Usually precipitated by a direct trauma or improper treatment treatment, spontaneous onset hematomas are rarely documented in the medical literature [1]. Although RSHs are more commonly found in older patients, age does not precipitate them. Patients with hemorrhagic shock generally display hemodynamic changes after losing 15% to 30% of their blood volume, according to widely accepted classifications. Despite the possibility in RSHs, only 1% to 13% of patients experience this. Although potentially lethal, the exact pathophysiology of a spontaneous burst is not well known. A misdiagnosis can often lead to imminent laparotomy with aggravated fatality [2]. Management depends on the patient’s condition, whether a conservative approach, angioembolization of the bleeding vessel, or surgery. Here, we present a 51-year-old male diagnosed with spontaneous RSH, which was managed by angioembolization of the bleeding vessel. However, the patient succumbed to acute respiratory distress syndrome (ARDS) later due to persistent hemorrhagic shock, which led onto cardiopulmonary failure in view of multiple organ dysfunction syndrome.

## Case presentation

A 51-year-old female presented with abdominal pain and constipation for about two days. Abdominal pain was insidious in onset, gradually progressive, and dull and had no relieving factors. She also complained of not passing stools for two days but passing flatus. The patient underwent a mechanical mitral valve replacement for rheumatic heart disease 10 years ago. She was on warfarin anticoagulation with low-dose aspirin after surgery for seven years and has stopped medication in the last three years. 

**Figure 1:**
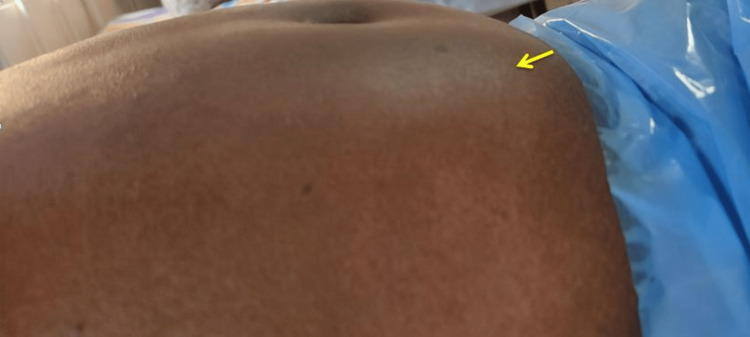
Clinical picture showing infraumbilical rectus sheath hematoma (arrow).

On examination, she was moderately built with severe pallor. The pulse rate was 90 beats/minute, and the blood pressure was 80/60 mm of Hg. The abdominal examination suggested a 3 cm x 3 cm RSH in the infraumbilical region (Figure 1).

There was lower abdominal distension with severe tenderness in the right iliac fossa, right flank region, and right thigh. A rectal examination showed hard stools without blood staining.

**Figure 2:**
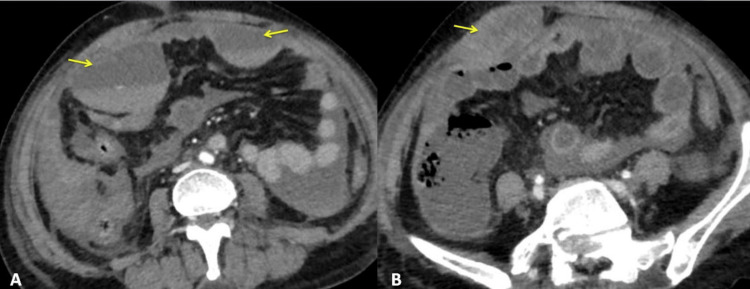
Computed tomography image (axial view) showing. A: Two well-defined hematomas in the right and left supra-umbilical region abutting the parietal peritoneum (arrows). B: The right rectus abdominis muscle appears bulky with an ill-defined hypodense rectus sheath hematoma (arrow).

Blood investigations were done, which revealed hemoglobin levels of 4 g/dl with normal liver function tests and renal function tests. The peripheral smear did not show hemolysis. The abdominal X-ray showed no air under the diaphragm or intestinal obstruction. Ultrasonography (USG) of the abdomen and pelvis showed a rectal sheath hematoma with moderate hemoperitoneum. Contrast-enhanced computed tomography (CT) with angiography of the abdomen and thorax revealed two well-defined hematomas in the right and left supra-umbilical regions abutting the parietal peritoneum, measuring 5 cm x 7 cm x 6.8 cm and 3.8 cm x 6.1 cm x 5.4 cm, respectively. A thin, hyperdense streak of contrast is noted within the right-sided hematoma in the arterial phase in the inferior aspect, probably leaking from the right inferior epigastric artery or the profunda femoris artery. The right rectus abdominis muscle appears bulky with an ill-defined hypodense RSH measuring 2 cm x 3.1 cm x 5.1 cm (Figure 2). 

**Figure 3:**
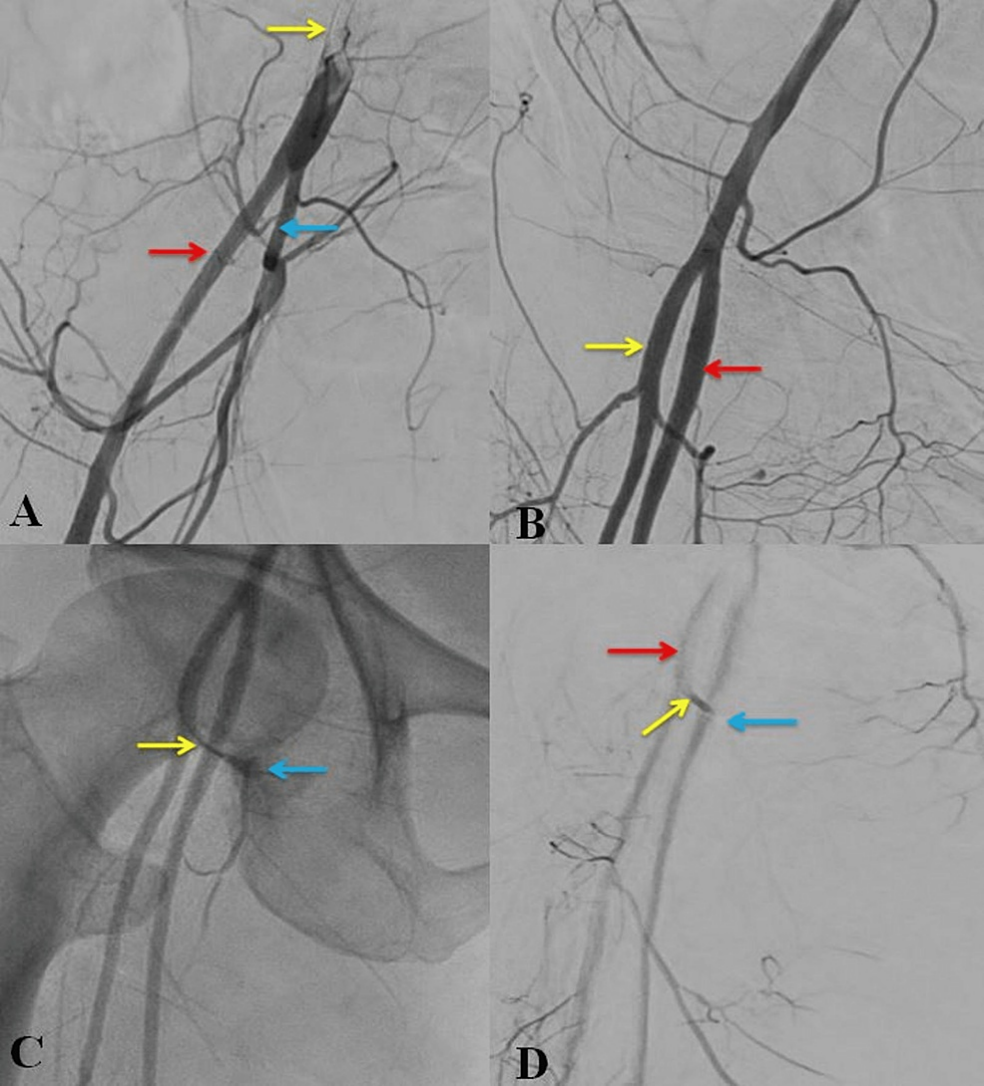
Digital subtraction angiography. A: Common iliac artery with a catheter inside (yellow arrow), external iliac artery (red arrow), and internal iliac artery (blue arrow). B: Superficial femoral artery (red arrow) and profunda femoris artery (yellow arrow). C: Catheter inside the branch of the profunda femoris artery (yellow arrow)which shows blush (blue arrow). D: Profunda femoris artery (red arrow) with a catheter inside the branch of it (yellow arrow) and employed branch without any more blush (blue arrow).

Warfarin toxicity was ruled out as the patient had not taken anticoagulants in the last three years and the international normalized ratio (INR) was 1.4. The patient was diagnosed with spontaneous RSH due to active bleeding from either the inferior epigastric artery or the profunda femoris artery. The patient was resuscitated with fresh frozen plasma (FFP), blood, platelets, and fluids. The patient was immediately taken up for digital subtraction angiography (DSA), and angioembolization of the branch of the right profunda femoris was done with polyvinyl alcohol (PVA) (Figure 3). Initially, the patient's vitals improved after embolization, but eventually she succumbed to ARDS.

Initially, the patient's vitals improved after embolization, but eventually, she succumbed to ARDS. This would have developed as a part of multiple organ dysfunction syndrome secondary to persistent shock. 

## Discussion

An RSH is a formation of blood within the rectus sheath that disrupts the rectus muscle or its blood supply, usually the inferior epigastric arteries [1]. It is a well-documented entity in the medical literature. However, all cases occur in the background of risk factors, such as anticoagulation therapy, post-traumatic bleeding, and post-surgical procedures [2]. In addition, it is seen in other cases, such as pregnancy, hypertension, chronic coughing, or liver disorders [2]. Patients often present with symptoms in the emergency setting, ranging from mild abdominal pain to life-threatening hemorrhagic shock. Although its association is with trauma and, in non-traumatic cases, with old age, coagulation disorders, or botched treatment, it can happen without an antecedent trigger, as in our case. The clinician needs a high level of suspicion to diagnose such a case when it presents, as it might harbor imminent lethality. The overall mortality in such cases have been historically seen to be around 4%, which steeply increases to 25% in the case of a patient undergoing anticoagulation [3].

It occurs more often in women than in men in the sixth to seventh decade of life [3]. The exact incidence and regulated trends have been difficult to estimate due to the sheer paucity of cases, but it has been reported in two studies in the past. Klingler et al. reported an incidence of 1.8% among 1857 patients, while Cherry et al. reported 1.6% among 126 patients in their respective studies [3]. RSHs are usually seen in the lower abdominal wall rather than the upper abdominal wall. This confers additional lethality owing to the absence of the sheath's tamponade effect. In addition, it has also been theorized that the acute, firm attachments of the vascular branches of the rectus as a muscle cause it to undergo shearing stress. This is in light of trauma or volume mediation. A single case report of a true spontaneous iliopsoas hematoma did not have any predisposing factors [1]. Our patient did not have any predisposing factors. The only factor that could have led to the catastrophic presentation was warfarin toxicity. However, our patient had stopped taking medications for three years.

Patients may present with features of an acute abdomen with or without peritonitis; a slow, tender, expansile abdominal mass; features of anemia; or features of hemorrhagic shock [4]. In rare cases, patients may also have confounding symptoms, such as fever, abdominal distension, gastrointestinal motility, and abdominal compartment syndrome. This is because of an enlarged intra-abdominal hematoma. Patients present with a tense, rigid abdomen with an apparent midline muscular hematoma. Ecchymoses, by default, develop mainly in the flanks and the periumbilical region, called Gray-Turner's and Cullen's signs. Our patient had abdominal pain and wall swelling along with RSH, without ecchymosis.

In rare cases of acutely expanding hematomas, intra-abdominal pressure might rise to a critical limit, warranting immediate decompressive laparotomy and surgical ligation of the bleeding vessel. In most cases, hematomas below the level of the arcuate ligament tend to be fatal because of an absent posterior rectus sheath. This prevents the occurrence of an internal tamponade. Our patient had an RSH on clinical examination, which was infraumbilical. There were no other palpable hematomas.

In their study, Berna et al. classified cases based on three types [5,6]. Type I are unilateral intramuscular hematomas that do not dissect fascial tension lines. Intramuscular type II can be bilateral but dissected along the fascial lines between the rectus and transversalis fascia. Type III includes hemoperitoneum, which usually advocates hemorrhagic shock and bears the highest prognostic mortality. The study classified the entity based on CT findings seen in 13 patients, which has led to a structured approach for these cases. The overall severity of the manifestation and end prognostication worsens as the type increases from 1 to 3. Our case was a type III RSH. 

RSH masquerades as acute abdomen-mimicking conditions, such as appendicitis, acute cholecystitis, diverticulitis, pancreatitis, and perforation peritonitis [3]. USG abdomen is usually the first investigation to diagnose the condition. Nevertheless, its sensitivity and specificity depend on the user, and technical difficulties in accessing the intermuscular planes and retroperitoneum eliminate its use as a test of the latter. A plain X-ray also helps rule out gut perforation. Angiography combined with CT of the abdomen has 100% diagnostic sensitivity, making it the diagnostic investigation of choice [7]. It enables you to identify the hematoma and the bleeding source. It also helps prognosticate the patient based on the Berna classification [7]. Furthermore, magnetic resonance imaging (MRI) can differentiate between chronic RSHs and abdominal wall tumors [8]. CT angiography reveals bleeding inferior epigastric artery or profunda femoris artery.

Overall mortality is approximately 4% in RSHs. Mortality is higher in anticoagulated patients but decreases with other risk factors. The Berna classification gives a layout of how most cases should be treated. Type I and type II rarely require hospitalization or blood resuscitation and usually recover within two to four months. Meanwhile, a type III patient would require intensive care unit (ICU) admission and strict hemodynamic monitoring, with an expected recovery time of four to six months [9].

In most cases, the algorithmic approach is based on clinical acumen and symptoms. Most cases respond to conservative clinical resuscitation, including blood transfusions, pain management, reversing coagulopathy, and blood pressure management. The critical aspect of this entity management is the low therapeutic threshold that the clinician should hold in response assessment. If the initial conservative approach does not show symptomatic improvements, patients are exposed to two invasive modalities. The first approach would be a DSA followed by therapeutic angiography with embolization of the bleeding vessel. Emergency laparotomy and ligation of the bleeding vessel will be required in hemodynamically unstable patients with abdominal compartment syndrome [10]. Invasive modalities have a high success rate, and recurrences have not been reported yet [10]. Our patient underwent successful angioembolization. However, the patient continued to deteriorate post-embolization and eventually passed away.

## Conclusions

In any event, RSH is an emergency requiring proper investigation and management. The decision for conservation or an invasive approach remains with the treating physician. An accurate diagnosis with prompt treatment and intervention helps rapid recovery and avoids poor outcomes. Patients with type III spontaneous RSH may deteriorate rapidly without immediate treatment. Even with rapid diagnosis and treatment, the patient may deteriorate and die.
